# Early Learning from the Healthy End of Life Project (HELP) Ottawa in the Context of COVID-19

**DOI:** 10.1093/geroni/igaa057.3528

**Published:** 2020-12-16

**Authors:** Pamela Grassau, Hayley Miloff, Emily Davison, Arne Stinchcombe, David Kenneth Wright, Roanne Thomas

**Affiliations:** 1 Carleton University, Ottawa, Ontario, Canada; 2 Brock University, St. Catharines, Ontario, Canada; 3 University of Ottawa, Ottawa, Ontario, Canada

## Abstract

Healthy End of Life Project (HELP) Ottawa is a community-based participatory research initiative which is based in four community sites in Ottawa, Ontario (Canada) (2 community health centers, and 2 faith communities). Focused on the needs of people who are frail, living with advanced illness, and their caregivers, including the needs of people who are bereaved, HELP Ottawa strives to, 1)strengthen informal and community social networks, organizational cultures and linkages across local health and social care services; 2)create a community culture that supports people to build social care networks to be able to ask for and accept help, and, 3)mobilize and prepare community members to be confident and capable of offering and providing help to people in their communities. Unfolding within the context of COVID-19, each HELP Ottawa site has found ways to mobilize, adapt and respond to lockdowns, quarantines, increased isolation and altered needs and services. Drawing on 89 initial consultations, followed by 111 interviews and 16 focus group participants (n=164), qualitative findings speak to the heightened grief and fear experienced within each site during the COVID-19 pandemic, and the multiple costs of severed ‘essential’ links. Critically highlighted is the need to build and sustain social supports and connection through everyday and local means while also integrating technology and online communication. Further apparent are the critical questions that need to be asked about how compassionate communities, and communities at large, can prepare for and respond to current and future waves of COVID-19.

